# Weighted Single-Step GWAS Identified Candidate Genes Associated with Growth Traits in a Duroc Pig Population

**DOI:** 10.3390/genes12010117

**Published:** 2021-01-19

**Authors:** Donglin Ruan, Zhanwei Zhuang, Rongrong Ding, Yibin Qiu, Shenping Zhou, Jie Wu, Cineng Xu, Linjun Hong, Sixiu Huang, Enqin Zheng, Gengyuan Cai, Zhenfang Wu, Jie Yang

**Affiliations:** 1 National Engineering Research Center for Breeding Swine Industry, College of Animal Science, South China Agricultural University, Guangzhou 510642, China; ruandl@stu.scau.edu.cn (D.R.); zwzhuang@outlook.com (Z.Z.); drr_scau@foxmail.com (R.D.); 13422157044qyb@gmail.com (Y.Q.); shenpingzhou1109@163.com (S.Z.); wujiezi163@163.com (J.W.); cnxu@stu.scau.edu.cn (C.X.); linjun.hong@scau.edu.cn (L.H.); sxhuang815@scau.edu.cn (S.H.); eqzheng@scau.edu.cn (E.Z.); cgy0415@163.com (G.C.); 2 Lingnan Guangdong Laboratory of Modern Agriculture, Guangzhou 510642, China

**Keywords:** Duroc pigs, growth traits, weighted single-step GWAS, SNP

## Abstract

Growth traits are important economic traits of pigs that are controlled by several major genes and multiple minor genes. To better understand the genetic architecture of growth traits, we performed a weighted single-step genome-wide association study (wssGWAS) to identify genomic regions and candidate genes that are associated with days to 100 kg (AGE), average daily gain (ADG), backfat thickness (BF) and lean meat percentage (LMP) in a Duroc pig population. In this study, 3945 individuals with phenotypic and genealogical information, of which 2084 pigs were genotyped with a 50 K single-nucleotide polymorphism (SNP) array, were used for association analyses. We found that the most significant regions explained 2.56–3.07% of genetic variance for four traits, and the detected significant regions (>1%) explained 17.07%, 18.59%, 23.87% and 21.94% for four traits. Finally, 21 genes that have been reported to be associated with metabolism, bone growth, and fat deposition were treated as candidate genes for growth traits in pigs. Moreover, gene ontology (GO) and Kyoto Encyclopedia of Genes and Genomes (KEGG) enrichment analyses implied that the identified genes took part in bone formation, the immune system, and digestion. In conclusion, such full use of phenotypic, genotypic, and genealogical information will accelerate the genetic improvement of growth traits in pigs.

## 1. Introduction

Pork is the primary source of protein for humans, with global pork consumption exceeding 110 metric kilotons per year [1]. Growth traits are economically important traits in porcine breeding programs, as accelerating the genetic process of growth-related traits can increase the supply of pork. At present, the age to 100 kg, average daily gain, backfat thickness, and lean meat percentage for a specific stage are vital indicators to measure the growth rate and carcass fat content of pigs due to their significant impact on production efficiency [2]. Furthermore, both genetic and non-genetic effects can affect growth traits, including pig breed, feeding behavior, and nutrition level. However, the above four traits have moderate heritability [3], suggesting that they could be improved by the genetic method.

Since the first genome-wide association study (GWAS) for age-related macular degeneration was published in 2005, GWAS has been widely used to identify quantitative trait loci (QTL) and to map candidate genes for complex traits in humans [4] and domestic animals [5]. Until now, 2036 QTL for growth traits have been reported in the pig QTL database (https://www.animalgenome.org/cgi-bin/QTLdb/SS/summary, release 27 August 2020). These findings have provided a certain number of molecular markers to porcine breeding for growth traits—for instance, Jiang et al. [6] performed a GWAS in a total of 2025 American and British Yorkshire pigs using PorcineSNP80 bead chip and detected five significant SNPs for days to 100 kg and the other five significant SNPs for 10th rib backfat thickness. Qiao et al. [7] found 14 QTL significantly associated with growth-related traits for White Duroc × Erhualian F2 and Sutai (Chinese Taihu × Western Duroc) populations. Although many studies have contributed to complex quantitative traits by GWAS, the genetic mechanisms of growth traits in pigs remain unclear. Additionally, some single marker GWAS analyses result in a weak power for QTLs detection and low accuracy for mapping. Moreover, most studies on GWAS for growth traits used the limited population size of genotyped animals and neglected the pedigree relationship. To overcome the limitation of the traditional GWAS approach, the weighted single-step GWAS (wssGWAS) proposed by Wang et al. [8] is preferable for livestock breeding, for which phenotypic and genealogical information is available for the vast majority of individuals and the small size of individuals genotyped.

The GWAS under the single-step genomic best linear unbiased prediction (ssGBLUP) framework is called ssGWAS, which intermixes genotypes, pedigree, and phenotypes data in a single analysis without creating pseudo-phenotypes [9]. However, when some traits are affected by significant QTL in practice, it is improper to account for all SNPs to explain the same proportion of genetic variance in ssGBLUP [10]. In that case, the wssGWAS can be adopted, which weighs SNPs according to their effects that were calculated genomic estimated breeding values (GEBVs) via ssGBLUP. The wssGWAS method has been successfully applied to detect supplementary QTLs and candidate genes in domestic and aquaculture animals, such as carcass traits in Nellore cattle [11], growth and carcass traits in rainbow trout [12], and reproductive traits in pigs [13]. However, to our knowledge, few wssGWASs have been performed to study growth traits in purebred Duroc pigs. Therefore, this study aims to identify genomic regions and candidate genes associated with growth traits such as days adjusted to 100 kg (AGE), average daily gain adjusted to 100 kg (ADG), backfat thickness (BF) and predicted lean meat percentage (LMP) adjusted to 100 kg in a Duroc pig population using the wssGWAS methodology. Then, gene ontology (GO) and Kyoto Encyclopedia of Genes and Genomes (KEGG) enrichment analysis facilitate further understanding of biological processes and functional terms of candidate genes for growth traits.

## 2. Materials and Methods

### 2.1. Ethics Statement

All animals used in this study were used according to the guidelines for the care and use of experimental animals established by the Ministry of Agriculture and Rural Affairs of China. The ethics committee of South China Agricultural University (SCAU, Guangzhou, China) approved the entire study. No experimental animals were anesthetized or euthanized in this study.

### 2.2. Animals, Phenotypes, and Pedigree

The animals used in this study were raised in two core farms of the Wens Foodstuff Group CO., Ltd. (Guangdong, China) with uniform standards. In brief, a total of 3945 Canadian Duroc pigs (1966 males and 1979 females) born between 2015 and 2017 were used in this study. Among them, 2084 individuals had genotypes and four growth-trait phenotypes in the pedigree, while 1843 ungenotyped individuals in the pedigree had phenotypes of AGE and ADG, and 1825 ungenotyped individuals in the pedigree had phenotypes of BF and LMP. Furthermore, the complete pedigree could be traced back 3 generations, with 5204 pigs in the full pedigree (2103 males and 3101 females).

Days to 100 kg and ADG were measured from 30 to 115 kg and then adjusted to 100 kg. AGE was adjusted to 100 kg using the formula below [14]:

(1) $$AGE\;adjusted\;to\;100\;\mathrm{kg}=\;Measured\;age-\left(\frac{Measured\;weight-100\;\mathrm{kg}}{Correction\;factor\;1}\right)$$

where the correction factor 1 of sire and dam are different, as follows:

(2) $$Sire:Correction\;factor\;1=\frac{Measured\;weight}{Measured\;age}\times 1.826$$

(3) $$Dam:Correction\;factor\;1=\frac{Measured\;weight}{Measured\;age}\times 1.715$$

*ADG* was adjusted to 100 kg by following formula [14]:

(4) $$ADG\;adjusted\;to\;100\;\mathrm{kg}=\frac{100\;\mathrm{kg}}{AGE\;adjusted\;to\;100\;\mathrm{kg}}$$

Adjusting LMP to 100 kg, phenotypes of BF and loin muscle depth (LMD) was measured between the last 3rd and 4th rib of Duroc pigs at the weight of 100 ± 5 kg by an Aloka 500 V SSD B ultrasound (Coromertics Medical Systems, USA) [15]. BF and LMD adjusted to 100 kg were calculated as reported by the Canadian Center for Swine Improvement (http://www.ccsi.ca/Reports/Reports_2007/Update_of_weight_adjustment_factors_for_fat_and_lean_depth.pdf):

(5) $$BF\;adjusted\;to\;100\;\mathrm{kg}=Measured\;BF\times \frac{A}{A+\left[B\times \left(Measured\;Weight-100\right)\right]}$$

where A and B are different for sire and dam, as follows:

(6) Sire:A=13.47; B=0.1115

(7) Dam:A=15.65; B=0.1566

*LMD* adjusted to 100 kg was calculated by the following equation [16]:

(8) $$LMD\;adjusted\;to\;100\;\mathrm{kg}=Measured\;LMD\times \left[\frac{a}{a+b\times \left(Measured\;Weight-100\right)}\right]$$

where a and b are gender-specific, and

(9) Sire:a=50.52; b=0.228

(10) Dam:a=52.01; b=0.228

*LMP* was adjusted to 100 kg using the formula below [16]:

(11) LMP adjusted to 100 kg=61.21920−0.77665×BF+0.15239×LMD

Overall, 3927 individuals were used in wssGWAS for ADG and AGE; 3909 individuals were used in wssGWAS for BF and LMP.

### 2.3. Genotyping and Quality Control (QC)

DNA was extracted from ear tissue of 2084 Duroc pigs following the standard phenol/chloroform method, then quantified and diluted to 50 ng/μL. All DNA samples were genotyped by GeneSeek porcine 50 K SNP chip from Illumina (Neogen, Lincoln, NE, USA), including 50,649 SNPs mapped to Sus scrofa11.1 (https://www.ensembl.org/biomart) in total. Quality control was performed by PLINK v1.09 (Boston, MA, USA) [17] in which SNPs were excluded when individuals call rate was <90%, SNPs call rate was <90%, Hardy–Weinberg equilibrium *p*-value was <10^−6^, minor allele frequency was <0.01, and SNPs were located in sex chromosomes and unmapped. After QC, a final set of 35,851 high-quality SNPs for 2084 Duroc pigs remained for subsequent analyses.

### 2.4. Statistical Analyses

Variance components for AGE, ADG, BF, and LMP traits were estimated with two methods using the average information restricted maximum-likelihood (AIREML), including pedigree-based Best Linear Unbiased Prediction (BLUP) and ssGBLUP. The four traits were analyzed using the same single-trait animal model, as described below:

(12) Y=Xb+Za+e

where Y was the vector of phenotypic values; *X* was the incidence matrix of fix effect for relating phenotypes; *b* was the vector of fixed effect, including birth year, sex, and farm; *Z* was the incidence matrix of random effect; a was the vector of additive genetic effects, and e was the vector of residuals. Narrow sense heritability was estimated as $h^{2}=\frac{\sigma_{a}^{2}}{\sigma_{a}^{2}+\sigma_{e}^{2}}$, where $\sigma_{a}^{2}$ and $\sigma_{e}^{2}$ were additive genetic variance and residual variance, respectively.

Additionally, the GEBVs of all individuals were estimated via the same single-trait model as described previously using the ssGBLUP [18] approach, and marker effects were calculated from the GEBVs. Comparing with the regular BLUP approach, ssGBLUP replaces the inverse of the pedigree relationship matrix ($A^{-1}$) with the matrix $H^{-1}$, for which the matrix H combined the pedigree and the genomic relationship matrices [19]. The inverse of matrix H was represented as follows:

(13) $$H^{-1}=A^{-1}+\left[\begin{array}{cc} 0 & 0\\ 0 & G^{-1}-A_{22}^{-1} \end{array}\right]$$

where $A_{22}^{-1}$ was the inverse matrix of the numerator relationship matrix considering genotyped animals and $G^{-1}$ was the inverse matrix of the genomic relationship matrix [20]. The genomic matrix *G* can be created as follows [21]:

(14) $$G=\frac{ZDZ^{\prime }}{\sum_{i=1}^{N}2p_{i}\left(1-p_{i}\right)}$$

where *Z* was a centered matrix of SNP genotypes (aa = 0, Aa = 1 and AA = 2), *D* was a matrix of weights for SNP variances, *n* was the number of SNPs and $p_{i}$ was the minor allele frequency of the i-th SNP [8].

The wssGWAS of SNP effects and weights were calculated following by Wang et al. [8]:

1. Initially, set $t=1,\;D_{\left(1\right)}=I$;
2. Calculate $G_{\left(t\right)}=\lambda ZD_{\left(t\right)}Z^{\prime }$, where $\lambda =\sum_{i=1}^{N}2p_{i}\left(1-p_{i}\right)$;
3. Calculate GEBVs for whole data set by ssGBLUP method;
4. Calculate SNPs effects: ${\hat{u}}_{\left(t\right)}=\lambda D_{\left(t\right)}Z^{\prime }G_{\left(t\right)}^{-1}\hat{g}$, where $\hat{g}$ was the GEBV of animals genotyped;
5. Calculate the weight of each SNP:
  (15) $$d_{i\left(t\right)}=2{\hat{u}}_{i\left(t\right)}^{2}p_{i}\left(1-p_{i}\right)$$
  where i was the i-th SNP;
6. Normalize SNP weights to keep total genetic variance constant via
  (16) $$D_{\left(t+1\right)}=\frac{tr\left(D_{\left(t\right)}\right)\times D_{\left(t+1\right)}}{tr\left(D_{\left(t+1\right)}\right)}$$
7. Set t=t+1, then loop to step 2.

The procedure was run for three iterations, as suggested by Wang et al. [8], which reached a high accuracy of GEBVs. In this study, SNPs located within 0.8 Mb (according to the linkage disequilibrium decay of this population [22]) were grouped in a window, and the percentage of genetic variance explained by each 0.8 Mb window was calculated following as below [8]:

(17) $$\frac{\mathrm{Var}\left(a_{i}\right)}{\sigma_{a}^{2}}\times 100\%=\frac{\mathrm{Var}(\sum_{j=1}^{x}Z_{j}g_{j})}{\sigma_{a}^{2}}$$

where $a_{i}$ was the genetic value of the *i*-th region consisting of x=0.8 Mb.

The procedures mentioned above were run with BLUPF90 software family programs [23] iteratively. The RENUMF90 module was used to obtain the required parameter file formats; the AIREMLF90 module was used for variance components estimation, the BLUPF90 module for GEBVs calculation, and the postGSF90 module for association analysis.

### 2.5. Identification of Candidate Genes and Functional Enrichment Analysis

Genomic windows that explained higher than 1.0% of the total genetic variance were selected as candidate QTL regions associated with growth traits in this study, which was also used in previous studies [8,13]. Since the 0.8 Mb window explained on–average 0.0473% (100% divided by 2115 genomic regions) of the genetic variance, the 1% threshold is over 20 times the expected average genetic variance explained by the 0.8 Mb window. The first three windows that explained the largest proportion of genetic variance for each trait were extended to 0.4 Mb flanking on either side of the regions. For the identified QTL regions, genes were searched using the Ensemble Sus scrofa 11.1 (https://www.ensembl.org/biomart) database within significant windows. To better understand the biological processes, GO and KEGG analyses were performed based on genes within significant regions using the database for annotation, visualization, and integrated discovery (DAVID v6.8, https://david.ncifcrf.gov/). A *p*-value of <0.05 was the threshold for significantly enriched GO terms and KEGG pathways.

## 3. Results and Discussion

### 3.1. Descriptive Statistics and Heritability for the Growth Traits

Descriptive statistics of the phenotypes are presented in Table 1. Previous studies reported that the average AGE phenotype of Duroc and other western commercial pig breeds was between 150 and 162 days, ADG was between 610 and 820 g/day, BF was between 11.69 and 18.19 mm, and LMP was between 56% and 62% [6,14,24,25,26]. The phenotypic averages for AGE, ADG, BF, and LMP in this study were similar to previous studies. The coefficients of variation (CV) for AGE, ADG, BF, and LMP were 7.30%, 7.25%, 17.86%, and 2.83%, respectively. The results indicated substantial phenotypic variation in these traits, except LMP. Since Duroc pigs are the terminal male parent of the Duroc × (Landrace × Yorkshire) pigs (DLY), the LMP of Duroc pigs receives long-term positive selection [27]. In other words, the lower CV of LMP indicates that the selection prior to the LMP was effective in this core Duroc population.

To better understand the genetic background of growth traits, we estimated the genetic variance ($\sigma_{a}^{2}$), residual variance ($\sigma_{e}^{2}$), and heritability ($h^{2}$) by different methods, including BLUP and ssGBLUP. The heritability estimated by BLUP and ssGBLUP were 0.507 and 0.343, 0.508 and 0.333, 0.512 and 0.315, and 0.554 and 0.332 for AGE, ADG, BF, and LMP, respectively (Table 2). There were differences in the heritability estimated by the two methods in this study, and the previous study showed that common environmental components lead to a possible overestimation of genetic variance in the pedigree-based BLUP method of estimating heritability [28]. Compared with the BLUP method, the ssGBLUP method has a lower standard error. The ssGBLUP method uses both pedigrees and genotyped information, and the estimated genetic parameters are theoretically more accurate [29]. Furthermore, the results from the two methods indicated that these traits were moderate heritability traits and could be genetically improved by genetic techniques.

### 3.2. Summary of wssGWAS

**Figure 1:**
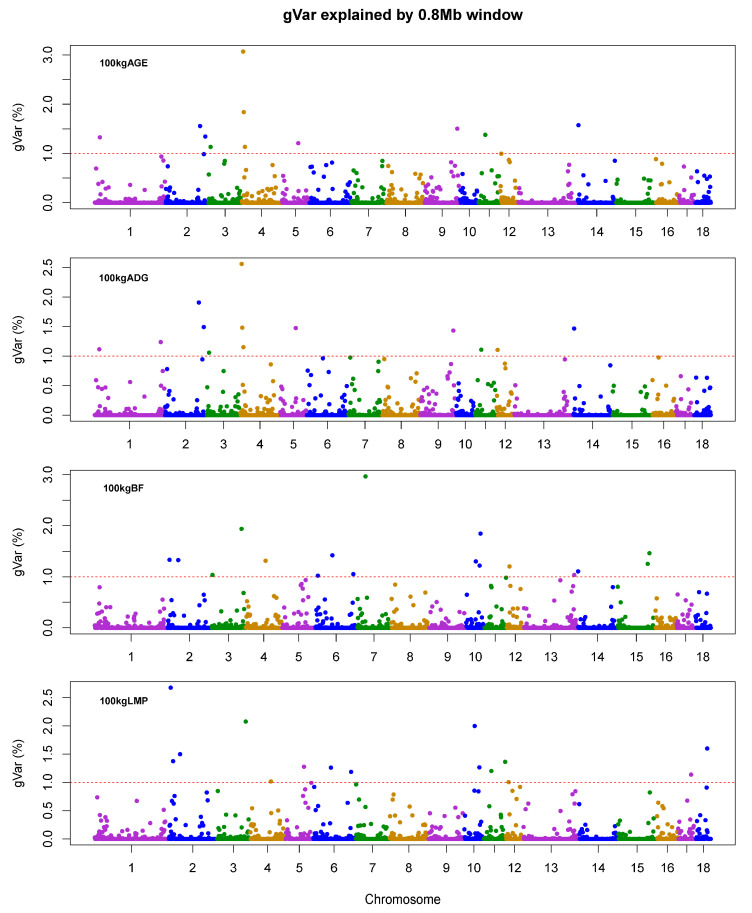
The proportion of genetic variances of the growth traits is explained by 0.8 Mb windows. gVar (%) represents the proportion of genetic variance explained by 0.8 Mb windows; 100 kg AGE, days to 100 kg; 100 kg ADG: average daily gain adjusted to 100 kg; 100 kg BF, backfat thickness adjusted to 100 kg; 100 kg LMP, predicted lean meat percentage adjusted to 100 kg.

Most important economic traits of livestock are quantitative traits with complicated genetic architectures. Therefore, uncovering the candidate genes underlying these traits has been a crucial goal in livestock breeding programs. In particular, growth rate and carcass fat content comprise the essential measuring basis of production performance in pigs, influencing the economic benefit directly. In this study, genetic variance explained by 0.8 Mb windows for each trait was achieved by wssGWAS. The first three most important QTL regions and the candidate genes are shown in Table 3. Overall, the first three QTL regions totally explained 5.96%–7.25% of the genetic variance of these traits under study. For each trait, the most significant windows explained approximately 2.56%–3.07% of the total genetic variance. Additionally, the identified windows (>1%) explained 17.07%, 18.59%, 23.87%, and 21.94% for AGE, ADG, BF, and LMP, respectively (Supplementary File, Tables S1–S5). Previous GWAS research reported that the candidate QTL regions of ADG on Sus scrofa chromosome (SSC) 1, 3, 6, 8, 13 and the candidate QTL regions of AGE on SSC 1, 3, 6, 8, 10, explaining a total of 8,09% and 4.08% of genetic variance [14], respectively. Due to LD, the wssGWAS method using the SNP window for analysis probably better identifies unknown QTL than the traditional GWAS, avoiding overestimation of the detected QTL number and false-positives [30,31]. Moreover, iterative weighting for SNPs could highlight QTL with larger effects [8]. Comparing with the results of the ssGWAS in ADG and BF by Matteo et al. [32], and our results identify the most significant QTL regions explaining greater genetic variance. Figure 1 shows the proportion of variance explained by each 0.8 Mb window for the studied traits, suggesting the polygenic genetic architecture of these traits.

### 3.3. wssGWAS for AGE and ADG

For AGE, 11 relevant QTL regions located on SSC 1, 2, 3, 4, 5, 9, 11, and 14 were identified (Supplementary Materials, Tables S1–S3). These regions explained 1.13–3.07% of total genetic variance for AGE, and 73 genes were annotated in these genomic regions. For ADG, 13 relevant QTL regions located on SSC1, 2, 3, 4, 5, 9, 11, 12, and 14 were identified, where 104 genes are located in these genomic regions (Supplementary Material, Tables S1 and S3). These regions explained total genetic variance ranged from 1.06% to 2.56% for ADG.

For the identified significant regions, there were 10 overlapped windows for AGE and ADG, which explained different proportions of genetic variance in these two traits. For complex quantitative traits, it was assumed that the linear effects of genes fitted the average of traits completely. However, the effects of genes are not always linear for the traits in practice, and the nonlinear assumption is more appropriate [14], which means that genes contributed differently and pleiotropic effects of the QTL between traits. QTLs with pleiotropic effects are common in the pig genome. For instance, Yang et al. [33] reported that a pleiotropic QTL on SSC 7 was associated with the vertebral number, carcass length, and teat number. In the present study, the region with the largest explained genetic variance for AGE and ADG, located in the region of 4.38–5.98 Mb on SSC4, seemingly had pleiotropic effects on meat and carcass traits in pigs [34]. Considering the duplication of identified windows and the strong genetic relationship of AGE and ADG, the genes identified by these two traits as common candidate genes are acceptable.

Among the significant windows of these two traits, the most important region (4.38–5.98 Mb on SSC4) harbored the *Family with Sequence Similarity 135 Member B* (*FAM135B*). The expression of *FAM135B* promotes granulin (GRN) secretion, and GRN is a secreted growth factor with high expression in epithelial, immune, chondrocytes, and neuronal cells [35]. Furthermore, *FAM135B* was reported as a candidate gene related to growth traits in beef cattle [36] and reproductive traits in Duroc pigs [37]. The *Zinc Finger And AT-Hook Domain Containing* (*ZFAT*) located in the region of 6.75–8.35 Mb on SSC4, and its mutation would lead to abnormal human body development and thyroid hormone secretion that played a key role in growth and metabolism [38].

The *Nuclear Factor, Interleukin 3 Regulated* (*NFIL3*) and the *Receptor Tyrosine Kinase-Like Orphan Receptor 2* (*ROR2*) were located in the regions of 1.63–3.23 Mb on SSC14. Wang et al. [39] reported that *NFIL3* affected the circadian lipid metabolism program, lipid–absorption, and export of intestinal epithelial through mouse experiments. The mice knocked out *ROR2* resulted in shortened or deformed bones and neurodevelopmental dysplasia [40].

The *Solute Carrier Family 27 Member 6* (*SLC27A6*) gene is located in the region of 130.75–132.35 Mb on SSC9. The *SLC27A6* gene had high expression in fat and muscle tissue and worked on lipid metabolism in pigs [41]. The *Adrenoceptor β 2* (*ADRB2*) gene, located in the region of 149.94–151.54 Mb on SSC2, encoded the β-adrenergic receptor that played an essential role in regulating metabolic level [42]. Furthermore, Bachman et al. [43] found that the knockout mice *ADRB*s have a reduced metabolic rate and accelerated fat deposition. The members of the *Tumor Necrosis Factor Receptor Superfamily* (*TNFS*), among which *TNFS11* was identified in the region of 24.24–25.04 Mb on SSC11, were responsible for bone growth in mice [44], and the variation of *TNFS11* led to the low level of serum insulin-like growth factor 1 (*IGF1*) influencing growth rate [45].

### 3.4. wssGWAS for BF

A total of 17 relevant QTL regions on SSC2, 3, 4, 6, 7, 10, 12, 13, 14, and 15 were identified for BF (Supplementary Material, Tables S1 and S4), where 99 genes were targeted in these genomic regions. These genomic regions explained 1.02–2.97% of the total genetic variance for BF.

The most significant window was located in the region of 29.34–30.94 Mb on SSC7, where four genes were targeted and were related to BF. In previous studies, *Death Domain Associated Protein* (*DAXX*) was reported to affect fat deposition and fatty acid synthesis via regulating the transcriptional activity of the androgen receptor negatively [46,47]. For *Inositol 1,4,5-Trisphosphate Receptor Type 3* (*ITPR3*), another gene located in the most important window, it was confirmed that mutations could cause taste disorders in mice [48]. Nonetheless, the *Inositol Hexakisphosphate Kinase 3* (*IP6K3*) gene was located in the same region. The mice without this gene resulted in a lower growth rate and metabolism and a shorter lifespan [49]. *Protein–Kinase C and Casein Kinase–Substrate In Neurons 1* (*PACSIN1*), a fourth gene located in the region of 29.34–30.94 Mb, was identified concerning the bodyweight [50] and loin muscle area [51] in pigs.

*CYP7A1*, a member of Cytochrome P450 Family 7 Subfamily A, was identified in the region of 74.12–74.92 Mb on SSC4. The *CYP7A1* gene-encoded enzyme cholesterol 7α-hydroxylase mainly catalyzes the decomposition of cholesterol and synthesis of cholic acid [52]. The *SECIS-Binding Protein 2* (*SECISBP2*) was located in the region of 0.43–1.22 Mb on SSC14, and its mutation brought about abnormal thyroid hormone metabolism in humans [53].

### 3.5. wssGWAS for LMP

Altogether, 15 relevant regions on SSC2, 3, 4, 5, 6, 10, 11, 12, 17 and 18 were identified for LMP. These regions explained 1.00–2.68% of total genetic variance for LMP and 115 genes located in these genomic regions (Supplementary Materials, Tables S1 and S5). The *N-α-Acetyltransferase 40* (*NAA40*) gene and *Galectin 12* (*LGALS12*) gene were located in the region of 8.11–9.71 Mb on SSC2 with the highest percentage of total genetic variance. Liu et al. [54] demonstrated that knockout male rats of the *NAA40* gene exhibited abnormal lipid metabolism and reduced fat mass. In addition, *NAA40* was identified to be associated with the metabolism/transport of fatty acids or lipids in pigs [55]. For *LGALS12*, this gene was preferentially expressed in adipocytes, and mice lacking *LGALS12* resulted in increased mitochondrial respiration, reduced adiposity and decreased insulin resistance/glucose tolerance [56]. Furthermore, *LGALS12* has been identified to be associated with intramuscular and subcutaneous fat in pigs [57].

The *Corticotropin-Releasing Hormone Receptor 2* (*CRHR2*) gene, located in the region of 42.05–42.83 Mb on 18, was highly expressed in adipose tissue, which was involved in the regulation of energy homeostasis and the anorexia effect of fat levels in the corticotropin-releasing hormone (CRH) system [58]. For the region of 41.40–42.12 Mb on SSC2, *Peroxisomal Biogenesis Factor 16* (*PEX16*) and *Cryptochrome Circadian Regulator 2* (*CRY2*) were associated with LMP. Hofer et al. [59] found that the silence of *PEX6* affects adipocyte differentiation and increases peroxisomal fatty acid oxidation–reduction. For the *CRY2* gene, Mármol-Sánchez et al. [60] reported that the polymorphism of *CRY2* was significantly associated with stearic acid content in the longissimus dorsi muscle in Duroc pigs. The *Acyl-CoA Thioesterase 8* (*ACOT8*) gene is located in the region of 47.67–48.83 Mb on SSC17. The protein encoded by this gene is an acyl-CoA thioesterase enzyme that influences the thyroid hormone to regulate lipid storage and utilization according to metabolic demands [61].

### 3.6. BF and LMP Overlap Regions

In the present study, six genomic regions were found to be associated with both BF and LMP, including 41.40–42.12 Mb on SSC2,117.76–119.36 Mb on SSC3, 67.38–68.18 Mb, and 155.99–156.71 Mb on SSC6, and 38.67–40.27 Mb and 55.95–57.55 Mb on SSC10. Notably, BF and LMP were used as an important indicator of carcass fat content in production. Moreover, the genetic correlation of lipid deposition with growth rate and feed efficiency traits were positively high and negatively moderate, respectively [62]. Therefore, these overlap and pleiotropic regions were valuable for growth traits in pigs.

*Potassium Inwardly Rectifying Channel Subfamily J Member 11* (*KCNJ11*), located in the region of 41.40–42.12 Mb on SSC2, was associated with type 2 diabetes in humans [63]. The region of 117.76–119.36 Mb on SSC3 was the second most important window for BF and LMP, which explained 1.94% and 2.08% of the additive genetic variance, respectively, and the *Syndecan 1* (*SDC1*) gene was detected. The *SDC1* gene has been proved to consume the intradermal fat layer, improve glucose tolerance, and significantly reduce body fat content in knockout mice [64]. Two genomic regions stood out on SSC6, which explained 1.05% and 1.42% of additive genetic variance for BF, and 1.26% and 1.19% of additive genetic variance for LMP, respectively. However, the annotated genes in one of these regions are not reported to be associated with growth traits, and no genes are described in the other region on SSC6, pending further studies.

The *Neuropilin 1* (*NRP1*) gene is located in the region of 55.95–57.55 Mb on SCC10, and several studies have exhibited its function in regulating fat cell–activity [65] and reducing dietary insulin resistance [66]. For the region of 38.67–40.27 Mb on SSC10, three genes were identified to be associated with BF and LMP. The *MOB Kinase Activator 3B* (*MOB3B*) gene was significantly associated with intramuscular fat and residual feed intake in cattle [67]. *Ras-Related Protein Rab-18* (*RAB18*), another gene located in the region of 38.67–40.27 Mb on SSC10, encoded a crucial Rab guanosine triphosphatase that controls the growth and maturation of lipid droplet, which lipid droplet was an intracellular organelle to stores triglycerides and cholesterol [68]. Still, in the same region, the *Membrane Palmitoylated Protein 7* (*MPP7*) gene was detected, and Bhoj et al. [69] reported that differences in *MPP7* gene expression affected glucose metabolism in the body.

### 3.7. GO and KEGG Analysis

In the current study, gene set enrichment analyses revealed that several terms might be related to growth traits. Among them, seven biological processes, two cellular components, one molecular function, and four KEGG pathways were targeted significantly (Table 4).

The positive regulation of bone mineralization (GO:0030501) is a key biological process of bone formation, which promotes the deposition of inorganic minerals in the organic–matter of the bone. Bone mineralization affects the strength and density of bone, enabling it to bear the body weight. Shim et al. [70] found that rapid weight gains were correlated with bone mineralization in broilers.

The positive regulation of lipophagy (GO:1904504) is an autophagic process that promotes cells to activate autophagy-related molecules to degrade lipids and regulate intracellular lipid content. Excessive fat deposition in pigs reduces feed conversion rate and affects growth rate, but also affects the quality of animal products [71]. Hence, the function of lipophagy in preventing excess fat deposition may improve the growth traits of pigs. Moreover, the PPAR signaling pathway (ssc03320) is the main pathway associated with lipid metabolism in pigs [72]. Free fat acid from lipophagy is a well-characterized ligand for PPARγ (peroxisome proliferator-activated receptor γ) [68], which activated the PPAR signaling to induce agouti-related peptide expression (AgRP). Sandoval et al. [73] found that AgRP co-expressed neuropeptide Y stimulated food intake and reduced energy expenditure. 

Potassium ion import (GO:0010107) mediates the transmembrane transport of ions and plays a key role in material exchange, energy transfer, and signal transduction. In particular, resting potassium currents make sour taste cells particularly sensitive to changes in intracellular pH, thereby affecting sour taste transduction [74]. Besides this, the taste transduction (ssc04742) pathway is the biological process by which the taste receptors of the organism detect and encode taste information through various transduction mechanisms. Several studies have shown that taste affects appetite and feed intake, and leads to a decrease in growth traits, such as body weight [75]. Moreover, the taste transduction pathway stimulates cephalic phase responses [76], promoting the process of salivary, gastric acid, and cephalic insulin secretion. Moreover, the insulin secretion (ssc04911) pathway was related to feeding intake, which promotes digestive metabolism and nutrient absorption and thus improves the growth trait.

## 4. Conclusions

In conclusion, we indicated 41 genomic regions to be associated with four growth traits (AGE, ADG, BF, and LMP) in a Canadian Duroc pig population using the wssGWAS method. The identified windows explained 1.00 to 3.07% of the genetic variance. Furthermore, 21 genes with related functional validation in previous studies were highlighted as candidate genes for growth traits in pigs. Moreover, GO, and KEGG enrichment analyses implied that the identified genes took part in bone formation, the immune system, and digestion, which were associated with growth traits. Such a full use of phenotypic and genotypic data and genealogical information will further advance our understanding of the genetic architecture and accelerate the genetic improvement of these economically important traits in pigs. In addition, the SNPs within identified regions may be useful for marker-assisted selection or genomic selection in future pig breeding.

## Figures and Tables

**Table 1:** Descriptive statistics of growth traits in the Duroc pig population.

Traits ^a^	*n*	Mean	SD ^b^	Min	Max	CV (%) ^c^
AGE	3927	163.41	11.93	125.98	206.32	7.30
ADG	3927	604.31	43.81	478.73	779.49	7.25
BF	3909	9.52	1.70	5.10	17.31	17.86
LMP	3909	61.08	1.39	54.93	65.06	2.28

^a^ AGE, days to 100 kg, ADG: average daily gain adjusted to 100 kg, BF, backfat thickness adjusted to 100 kg; LMP, predicted lean meat percentage adjusted to 100 kg; ^b^ SD, standard deviation; ^c^ CV, coefficient of variation.

**Table 2:** Variance components and heritability estimates of growth traits.

Traits ^a^	Models	$\sigma_{a}^{2}$ *	$\sigma_{e}^{2}$ *	$\sigma_{p}^{2}$ *	$h^{2}$ (SE) *
AGE	BLUP	68.667	66.879	135.546	0.507 (0.0454)
	ssGBLUP	44.932	85.981	130.913	0.343 (0.0314)
ADG	BLUP	926.290	895.570	1821.860	0.508 (0.0453)
	ssGBLUP	581.4	1166.3	1747.7	0.333 (0.0308)
BF	BLUP	1.516	1.445	2.961	0.512 (0.0449)
	ssGBLUP	0.877	1.903	2.780	0.315 (0.0289)
LMP	BLUP	1.142	0.918	2.060	0.554 (0.0444)
	ssGBLUP	0.639	1.283	1.922	0.332 (0.0289)

^a^ AGE, days to 100 kg, ADG: average daily gain adjusted to 100 kg, BF, backfat thickness adjusted to 100 kg; LMP, predicted lean meat percentage adjusted to 100 kg; * $\sigma_{a}^{2}$, genetic variance, $\sigma_{e}^{2}$, residual variance, $\sigma_{p}^{2}$, phenotypic variance, $h^{2}$, heritability; SE, standard error.

**Table 3:** First three most important quantitative trait loci (QTL) regions and candidate genes for growth traits.

Traits ^a^	Chr ^b^	Position (Mb)	nSNPs	gVar (%) ^c^	Candidate Genes
AGE	4	4.38–5.98	43	3.07	*FAM135B*
	4	6.75–8.35	43	1.84	*ZFAT*
	14	1.63–3.23	22	1.57	*NFIL3, ROR2*
ADG	4	4.38–5.98	43	2.56	*FAM135B*
	2	130.75–132.35	20	1.91	*SLC27A6*
	2	149.94–151.54	29	1.49	*ADRB2*
BF	7	29.34–30.94	26	2.97	*DAXX, ITPR3, IP6K3, PACSIN1*
	3	117.76–119.36	19	1.94	*SDC1*
	10	55.95–57.55	29	1.85	*NRP1*
LMP	2	8.11–9.71	26	2.68	*NAA40*, *LGALS12*
	3	117.76–119.36	39	2.08	*SDC1*
	10	38.67–40.27	15	2.00	*MOB3B*, *RAB18*, *MPP7*

^a^ AGE, days to 100 kg, ADG: average daily gain adjusted to 100 kg, BF, backfat thickness adjusted to 100 kg; LMP, predicted lean meat percentage adjusted to 100 kg; ^b^ Chr, chromosome; ^c^ gVar (%) represents the proportion of genetic variance explained by 0.8 Mb. For each trait, the genomic regions are sorted in descending order according to the proportion of genetic variance explained.

**Table 4:** Significant gene ontology (GO) terms and Kyoto Encyclopedia of Genes and Genomes (KEGG) pathways associated with growth traits in Duroc pigs (*p* < 0.05).

Term ^a^	Count	*p*-Value	Genes
GO:0003727—single-stranded RNA binding	4	0.004495	*SNRPC*, *NXF1*, *JMJD6*, *POLR2G*
GO:0032435—negative regulation of proteasomal ubiquitin-dependent protein catabolic process	3	0.020544	*WAC*, *UBXN1*, *SDCBP*
GO:0002924—negative regulation of humoral immune response mediated by circulating immunoglobulin	2	0.029686	*PTPN6*, *FOXJ1*
GO:0030335—positive regulation of cell migration	5	0.031742	*ROR2*, *SEMA4D*, *CSF1R*, *SDCBP*, *SPHK1*
GO:0008076—voltage-gated potassium channel complex	4	0.035714	*KCNC1*, *KCNJ11*, *KCNJ2*, *ABCC8*
GO:0005783—endoplasmic reticulum	11	0.038199	*GPC2*, *CREB3L1*, *VWF*, *P3H3*, *BRINP1*, *ATL3*, *PLAAT3*, *EEF1G*, *SRP68*, *CLDN14*, *GANAB*
GO:1904504—positive regulation of lipophagy	2	0.044199	*ADRB2*, *SPTLC1*
GO:0032651—regulation of interleukin-1 β production	2	0.044199	*S1PR3*, *SPHK1*
GO:0030501—positive regulation of bone mineralization	3	0.049487	*ADRB2*, *OSR1*, *FBN2*
GO:0010107—potassium ion import	3	0.049487	*KCNJ11*, *KCNJ16*, *KCNJ2*
ssc04742—taste transduction	5	0.000381	*TAS1R1, GRM4, ITPR3, GNB3, SCNN1A*
ssc04911—insulin secretion	5	0.019468	*CREB3L1, KCNJ11, CAMK2A, ITPR3, ABCC8*
ssc04725—cholinergic synapse	5	0.045538	*CREB3L1*, *CAMK2A*, *ITPR3*, *GNB3*, *KCNJ2*
ssc03320—PPAR signaling pathway	4	0.047474	*ACOX1*, *SLC27A6*, *PLTP*, *CYP7A1*

^a^ GO, gene ontology, KEGG, Kyoto Encyclopedia of Genes and Genomes pathway.

## Data Availability

The datasets generated and/or analyzed during the current study are not publicly available since the studied population is consisted of the nucleus herd of Wens Foodstuff Group Co., Ltd., but are available from the corresponding author on reasonable request.

## Supplementary Materials

The following are available online at https://www.mdpi.com/2073-4425/12/1/117/s1, Table S1: Genomic regions of 0.8 Mb explained more than 1% of genetic variance for growth traits in Duroc pigs; Table S2: The explained genetic variance of SNPs within significant windows for AGE; Table S3: The explained genetic variance of SNPs within significant windows for ADG; Table S4: The explained genetic variance of SNPs within significant windows for BF; Table S5: The explained genetic variance of SNPs within significant windows for LMP.
